# Intrauterine administration of peripheral blood mononuclear cells helps manage recurrent implantation failure by normalizing dysregulated gene expression including estrogen-responsive genes in mice

**DOI:** 10.1186/s12964-024-01904-3

**Published:** 2024-12-05

**Authors:** Yoshimi Kitawaki, Akihito Horie, Asami Ikeda, Shimpei Shitanaka, Akihiro Yanai, Tsutomu Ohara, Baku Nakakita, Yusuke Sagae, Asuka Okunomiya, Hirohiko Tani, Masaki Mandai

**Affiliations:** 1 Department of Gynecology and Obstetrics, Kyoto University Graduate School of Medicine, 54 Shogoin Kawahara-Cho, Sakyo, Kyoto, 606-8507 Japan; 2 Department of Gynecology and Obstetrics, Medical Research Institute Kitano Hospital, 2-4-20 Ohgimachi, Kita-Ku, Osaka, 530-8480 Japan; 3 Department of Gynecology and Obstetrics, Shizuoka General Hospital, 4-27-1 Kita Ando Aoi-Ku, Shizuoka, 420-8527 Japan

**Keywords:** Peripheral blood mononuclear cell, Intrauterine PBMC therapy, Recurrent implantation failure, Human chorionic gonadotropin, Embryo implantation, RNA-sequencing, Estrogen signaling

## Abstract

**Background:**

Intrauterine peripheral blood mononuclear cell (PBMC) therapy for recurrent implantation failure (RIF) has been reported to improve embryo implantation by acting on the endometrium. However, the exact mode of action of PBMC on the endometrium of patients with RIF remains unclear. In addition, the differences in the therapeutic effects of PBMC therapy with and without human chorionic gonadotropin (hCG) are unknown. Therefore, in this study, we investigated the changes in the endometrium during the implantation phase induced by PBMC administration and the differences in the efficacy of this therapy with and without hCG using a mouse model of implantation failure (IF).

**Methods:**

IF model was established by the subcutaneous administration of low-dose RU486. Pregnant mice were randomly divided into five groups: control, IF, culture medium, PBMC, and PBMC-hCG (the latter three groups were IF model mice with intrauterine administration). The pregnancy rate and the number and size of implantation sites were recorded during early pregnancy (day 7.5). Uteri from the preimplantation phase (evening of day 3.5) were collected and analyzed using RNA-sequencing (RNA-seq).

**Results:**

The pregnancy rate, the number of implantation sites, and the number of normal-sized implantation sites were significantly decreased in the IF model and were improved in the medium, PBMC, and PBMC-hCG groups. RNA-seq data showed that PBMC treatment normalized the expression of the majority of dysregulated genes in the endometrium during the preimplantation phase in the IF model, especially the overexpression of estrogen-activated genes. In addition, PBMC treatment increased the expression of local glucocorticoid receptors and suppressed the expression of inflammation-related genes, whereas no significant changes in blood estradiol and glucocorticoid levels were observed. These changes were more pronounced in the PBMC-hCG group and were consistent with the pregnancy outcomes.

**Conclusions:**

Intrauterine administration of PBMC before embryo implantation promoted embryo implantation in the IF mouse model, and hCG enhanced pregnancy outcomes. PBMC modulated steroid receptor expression and suppressed inflammation and excessive estrogen action.

**Supplementary Information:**

The online version contains supplementary material available at 10.1186/s12964-024-01904-3.

## Background

Despite the developments in in vitro fertilization (IVF) techniques, several individuals still face issues with conception. Recurrent implantation failure (RIF) is defined as the successive failure to achieve clinical pregnancy after three or more transfers of morphologically favorable embryos [1]. The causes of RIF are diverse, including factors related to embryo such as aneuploidy; gametogenic factors such as sperm and egg quality; uterine factors such as anatomic lesions or chronic endometritis; and abnormal immune tolerance [2, 3]. Although various treatments for RIF are available, targeting immune cell or endometrial functional abnormalities in RIF remains unexplored.

Various studies have shown that immune cells and hormonal control of the endometrial environment are crucial for embryo implantation [4, 5]. Several therapies targeting the immune system have been employed to treat RIF, and we developed an autologous intrauterine peripheral blood mononuclear cell (PBMC) therapy [6] and have performed it to date. In a preliminary study, PBMCs promoted the invasion and spread of BeWo cells and mouse blastocysts, which was enhanced by human chorionic gonadotropin (hCG). hCG promotes IL-8 production by PBMCs, and IL-8 promotes embryo invasion [7, 8]. Based on these findings, treatment protocols using PBMCs cultured with hCG have been performed as follows: PBMCs isolated from patients were cultured with hCG and administered into the uterus prior to embryo transfer. Randomized controlled studies and meta-analyses have already shown that PBMC therapy improves implantation and live birth rates in patients with RIF [9–12].

RU486 (mifepristone), an antagonist of progesterone and glucocorticoid receptors (GRs), induces ultrastructural changes in the endometrium [13]. In mice, a single dose of RU486 administered before embryo implantation alters endometrial morphology and inhibits pregnancy [14]. Intrauterine administration of PBMCs improves pregnancy outcomes in RU486-treated mice [15], and hence, this agent has been used in basic studies on PBMC therapy. Chen et al. and Rezaee et al. reported that PBMC administration improves gene expression profiles and embryo attachment rate to the endometrium in mouse models, respectively, suggesting that PBMCs positively affect endometrial function [16, 17]. A study using human endometrial epithelial cells also suggested that PBMCs affect the endometrium and promote embryo attachment [18]. However, the mechanisms explaining the influence of PBMC therapy on the endometrium remain unexplained. Moreover, the contribution of hCG treatment of PBMCs to the efficacy of this therapy remains unclear.

Herein, we aimed to investigate (i) the mechanism by which PBMC intrauterine administration improves endometrial abnormalities and (ii) the effect of hCG supplementation on therapeutic efficacy in vivo. We compared the pregnancy outcomes of PBMC intrauterine administration with or without hCG in a mouse model of implantation failure that was induced by RU486 and analyzed gene expression in the implantation-phase endometrium.

## Methods

### Animals

B6D2F1 mice (Japan SLC, Inc., Shizuoka, Japan) were maintained in a controlled environment (24 °C, 50% relative humidity, 14-h light/10-h dark). All animal experiments were approved by the Animal Research Committee of Kyoto University (Med Kyo 21,135) and conducted according to the guidelines of the Science Council of Japan.

### Experimental design

**Fig. 1:**
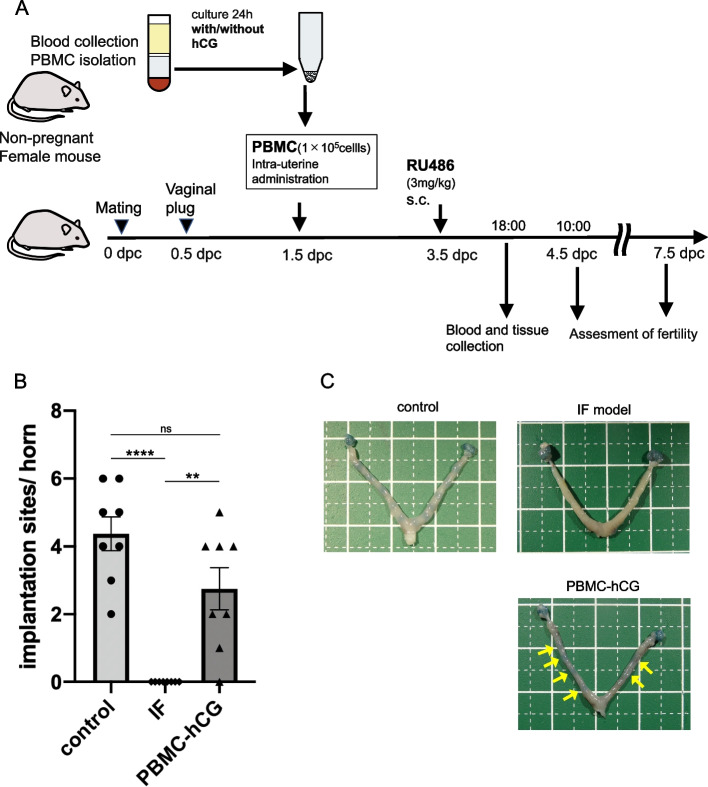
Timeline of the experimental procedure and pregnancy outcome at 4.5 dpc. **a** PBMCs were collected from non-pregnant female mice and cultured with or without hCG for 24 h. PBMCs were concentrated and administered into the uterine cavities of pregnant mice at 1.5 dpc. Pregnant mice were injected with 3 mg/kg RU486 at 3.5 dpc, to establish a model of implantation failure. The uterus and blood were collected in the evening (18:00) at 3.5 dpc before the initiation of embryo attachment for RNA extraction, HE staining, and ELISA. Embryo implantation was assessed at 4.5 dpc morning (10:00) or 7.5 dpc. **b** Average number of ISs per horn in control (normal pregnant) and IF model mice untreated or treated with PBMC (cultured with hCG) at 4.5 dpc (*n* = 8 in each group). ** *p* < 0.01, **** *p* < 0.0001, ns *p* ≥ 0.05. IF: IF model mice, PBMC-hCG: IF model mice treated with intrauterine PBMC. **c** Representative uteri in the control, IF, and PBMC-hCG group at 4.5 dpc. Arrows indicate implantation sites marked with Chicago Blue dye solution. dpc = days post coitum, IS = implantation site

Female mice (age: 8–12 weeks) in the estrous cycle were mated with males. The morning of the vaginal plug was designated as day 0.5 post coitum. An implantation failure (IF) model was established by the subcutaneous administration of low-dose RU486 (3 mg/kg) at 9:00 AM on day 3.5, based on previous reports [15]. On the morning of day 4.5, the mice were sacrificed by cervical dislocation under anesthesia, and the implantation sites were detected using intravenous Chicago Blue injection to confirm implantation failure at the early stage of implantation in the model. In subsequent experiments, pregnant female mice were randomly assigned to control, IF, medium, PBMC, and PBMC-hCG groups (the latter three groups were the IF model with each treatment) for fertility assessment and sample collection (Fig. 1a).

### PBMC isolation

PBMCs were isolated from non-pregnant mice by density gradient centrifugation using Lympolyte-M (Cedarlane Laboratories, Burlington, ON, Canada). PBMCs were collected from the middle layer, subjected to hemolysis (Pharm Lyse lysing buffer; BD Biosciences, San Jose, CA, USA), and washed twice with PBS. PBMCs (1 × 10^6^ cells/mL) were suspended in RPMI1640 (Nacalai Tesque, Kyoto, Japan) + 10% FBS (Sigma-Aldrich, St. Louis, MO, USA) + penicillin–streptomycin 100 U/mL (Gibco, Thermo Fisher Scientific, Waltham, MA, USA) and then incubated with or without hCG (5 IU/mL, Mochida Pharmaceutical CO, Tokyo, Japan) for 24 h at 37°C, 5% CO_2_.

### Intrauterine administration of PBMCs

Cultured PBMCs were collected by pipetting, and the supernatant was removed using centrifugation. Subsequently, these PBMCs were resuspended in a culture medium, and 2.5 μL of cell suspension (0.5 × 10^5^ cells) was injected into each bilateral uterine horn using an embryo manipulation glass pipette to PBMC and PBMC-hCG groups on day 1.5. The medium group was administered only the culture medium containing hCG. These procedures were performed under anesthesia.

### Assessment of pregnancy outcome and tissue collection

On day 7.5, mice in each group were sacrificed, the number and size of implantation sites were recorded, and the uteri were collected. The uterine horns were fixed in 4% paraformaldehyde (PFA), embedded in OCT, and flash-frozen for histological analysis. Another set of mice was sacrificed on the evening of day 3.5 (18:00). One horn was immediately placed in RNA*later* (Invitrogen, Thermo Fisher Scientific) for RNA extraction, and the other horn was fixed in 4% PFA and embedded in OTC for histology, then flash-frozen at -20 and -80 °C. Blood was collected from some mice on the evening of day 3.5 before sacrifice, the plasma was isolated, quick-frozen, and stored at -80°C until use. Mice on the morning of day 3.5 (9:30) were also sacrificed and their uteri were collected for histological analysis.

### Hematoxylin–eosin (HE) staining and immunohistochemistry

Frozen sections of mouse uterine tissue were prepared as previously described [19, 20] and stained with HE. Images of implantation sites were captured using an optical microscope (BX51 and DP-20; Olympus, Tokyo, Japan). Immunostaining for Ki-67 expression was performed using antibodies; the method is presented in Table S1 (Additional file 1) and Additional file 2.

### RNA extraction

RNA was extracted from the uterine tissues using the Invitrogen PureLink RNA mini kit (Thermo Fisher Scientific) according to the manufacturer's instructions and treated with DNase I to remove genomic DNA contamination. RNA quality (RIN ≥ 9) was confirmed using Agilent Bioanalyzer 2100 (Agilent Technologies, USA).

### RNA-sequencing and transcriptome data analysis

Libraries were prepared from the total RNA extracted from uterine tissue on the evening of day 3.5 in each group (*n* = 5) using the TruSeq Stranded mRNA LT Sample Prep Kit (Illumina, San Diego, CA, USA) and sequenced on a NovaSeq 6000 (Illumina). The reads were trimmed using the Trimmomatic tool [21] and mapped to the reference genome (mm10) with HISAT2 [22]. Then, transcript assembly was performed using Stringtie [23]. Differentially expressed gene (DEG) analysis was performed using EdgeR [24]; read count data were normalized using the trimmed mean of M-value (TMM) method for genes with read counts greater than 0 in all samples and tested using exactTest. Genes satisfying |fold change|≥ 2 and exact raw *p*-value < 0.05 were designated as DEGs.

Gene ontology (GO) analysis was performed using the gProfiler web tool (https://biit.cs.ut.ee/gprofiler/), and upstream regulator analysis was performed using the Ingenuity Pathway Analysis software (Qiagen, Hilden, Germany). Estrogen-responsive genes in the mouse uterus were defined from the DEGs (|fold change|≥ 2, false discovery rate < 0.1) in the uterus after 2 and 6 h of estrogen administration in ovariectomized mice (GSE133158) [25]. See Additional file 2 for more details on the methods.

### Quantitative real-time PCR

cDNA was prepared from the total RNA of each sample using ReverTra Ace qPCR RT Master Mix (TOYOBO, Osaka, Japan). Real-time quantitative PCR (qPCR) was performed using the THUNDERBIRD SYBR qPCR Mix (TOYOBO) and Applied Biosystems StepOnePlus (Thermo Fisher Scientific). RNA expression values were corrected against *Gapdh* and *Rplp0* expression levels, and relative expression levels were calculated using the 2-ΔΔCt method [26]. The primers used for qPCR are listed in Table S2 (Additional file 1).

### Hormone assay (ELISA)

Plasma estradiol and corticosterone concentrations on the evening of day 3.5 were measured using a 17β-Estradiol high-sensitivity ELISA kit and a Corticosterone ELISA kit (Enzo Life Sciences, Farmingdale, NY, USA), respectively, according to the manufacturer's instructions.

### Statistical analyses

Comparative analyses of implantation sites and qPCR results were performed using one-way analysis of variance (ANOVA) followed by Tukey’s and Sidak’s post-hoc multiple comparison methods, respectively. The pregnancy rates for each group were tested using the Fisher’s exact test, and p-values were corrected for multiple testing using the Benjamini–Hochberg method. Statistical analyses were performed using the GraphPad Prism 9 software (GraphPad Software, Boston, MA, USA). Data are presented as the mean ± SEM unless otherwise noted.

## Results

### Validation of the IF model on the morning of day 4.5

In contrast to that in the control group, no implantation sites were detected in the uteri of IF model mice on the morning of gestation day 4.5, the early stage of embryo implantation. However, in the IF model mice treated with hCG-supplemented PBMC (PBMC-hCG group), implantation sites were detected in most of the uteri (Fig. 1b, c). This confirmed that embryo implantation was impaired at an early stage in the IF model and was rescued by PBMC-hCG treatment. There were no obvious morphological differences in the uterus between the IF and control groups on day 3.5, immediately before implantation; PBMC-hCG administration did not induce morphological changes (Fig. S1, Additional file 3).

### Pregnancy outcome on day 7.5

To examine the effect of intrauterine administration of PBMC on embryo implantation, we compared the pregnancy rate, number of implantation sites, and implantation site size of the plug-positive mice in each group on day 7.5.

**Fig. 2:**
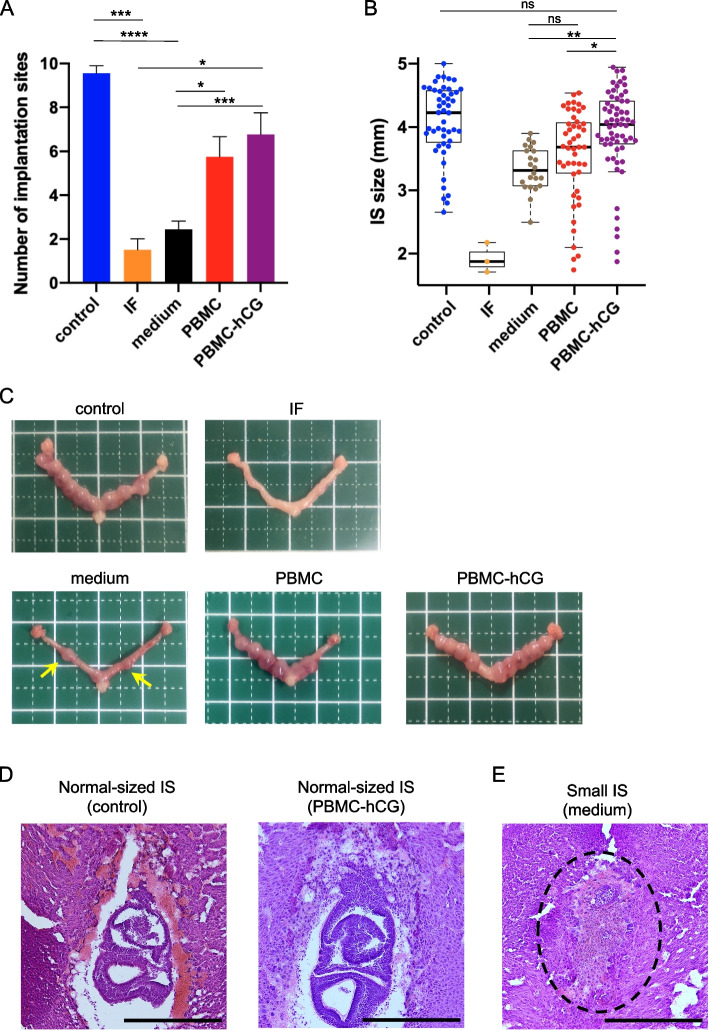
Embryo implantation in each group at 7.5 dpc. Intrauterine PBMC administration improves implantation site (IS) number and reduces abnormally sized IS at 7.5 dpc. **a** Average number of IS per mouse in each group at 7.5 dpc (*n* = 2–9 per group). **b** Diameter of implantation sites at 7.5 dpc (*n* = 2–9 per group). Box plots show the median (bold horizontal line), interquartile range (box), and range of values, excluding outliers (whiskers). * *p* < 0.05, ** *p* < 0.01, *** *p* < 0.005, **** *p* < 0.0001, ns *p* ≥ 0.05. **c** Representative uteri of each group. Arrows indicate small-sized IS. **d**,** e** Representative hematoxylin and eosin-stained sections of normal- or abnormally sized implantation sites. Abortion (embryo resorption) images showing hemorrhage and loss of normal embryos observed at small implantation sites. Black circles indicate the abortion sites. The scale bar represents 500 µm

The pregnancy rate in the IF group was significantly lower than that in the control group (*p* < 0.01), whereas the pregnancy rates in the medium, PBMC, and PBMC-hCG groups were higher than those in the IF group (*p* < 0.05; Table 1). The number of implantation sites was also significantly lower in the IF group than in the control group (*p* < 0.001) and improved to different degrees in each treatment group compared to the IF group. The strongest effect was observed in the PBMC-hCG group, with a significant increase in the number of implantation sites compared to that in the medium and control groups (*p* < 0.05, *p* < 0.001, respectively; Fig. 2a). The PBMC group also had a significantly increased number of implantation sites compared to that in the medium group (*p* < 0.05; Fig. 2a). The size of the implantation sites in pregnant mice was significantly larger in the PBMC-hCG group than in the medium and PBMC groups (*p* < 0.01 and *p* < 0.05, respectively) and was comparable to that of the control group (Fig. 2b, c).

**Table 1:** Pregnancy rate of mice with a copulation plug in each group on day 7.5 post coitum

		**Treatment**			
**Group**	**RU486**	**hCG**	**PBMC**	**N**	**Pregnancy rate (%)**
Control	-	-	-	10	90
IF	+	-	-	13	15.3 **a
Medium	+	+	-	13	69.2 *b
PBMC	+	-	+	13	61.5 *b
PBMC-hCG	+	+	+	13	69.2 *b

The pregnancy rate was significantly higher in the medium, PBMC, and PBMC-hCG groups than in the IF group

*n* = 10–13 in each group

^**^a *p* < 0.01 compared with control group, *b *p* < 0.05 compared with IF group

The Fisher’s exact test was used for comparison, and *p*-values were corrected for multiple testing using the Benjamini–Hochberg method

### Histology of implantation site on day 7.5

HE staining of the implantation site on day 7.5 revealed that normal-sized implantation sites (> 3.5 mm) in the PBMC-hCG group contained normal fetuses equivalent to those at 7.5 days of gestation in normal pregnant mice. By contrast, small implantation sites (approximately 3 mm) observed in the three groups (PBMC-hCG, PBMC, and medium), and especially in the medium group, contained small fetuses equivalent to 5.5–6.5 days of gestation or did not contain fetus, indicating abortion (Fig. 2d, e). In the PBMC group, the histology varied depending on the size of the implantation site, with a higher number of small fetuses than in the PBMC-hCG group and abortion findings similar to those in the medium group (Fig. S2a, Additional file 4). Histology of the IF group showed no fetus, consistent with the findings of no implantation site (Fig. S2b, Additional file 4).

### Transcriptomic changes by PBMC administration in the uterus at preimplantation period (evening of day 3.5)

**Fig. 3:**
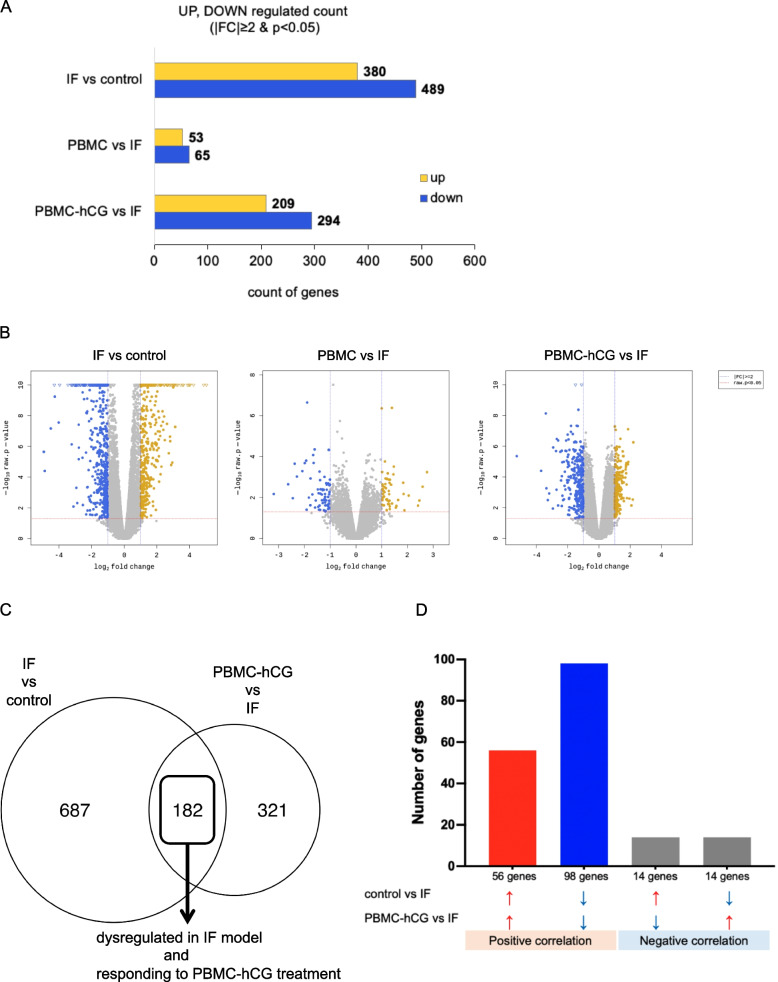
RNA analysis of uteri at 3.5 dpc; number of differentially expressed genes (DEGs) identified in each comparison. Administration of hCG-treated PBMCs rescued the dysregulated gene expression in the uteri of the IF model. **a**, **b** Number of DEGs and volcano plots of IF versus control, PBMC versus IF, and PBMC-hCG versus IF. **c** Venn diagrams showing the overlap of all DEGs between the IF versus control and PBMC-hCG versus IF groups. **d** Graph depicting the direction of gene expression changes in the 182 overlapping DEGs between the control versus IF and PBMC-hCG versus IF comparisons. DEGs were identified as *p* < 0.05 and log2(fold change) ≥ 1

To identify the molecular mechanism by which intrauterine administration of PBMC rescues embryo implantation in the IF model, we performed transcriptome analysis of endometrium on the evening of day 3.5, just before embryo implantation, for four groups: control, IF, PBMC, and PBMC-hCG. A total of 869 DEGs (380 upregulated and 489 downregulated genes; identified by |fold change| ≥ 2 and *p* < 0.05) were detected in the IF group when compared to the control group. In the PBMC-hCG group, 503 DEGs were detected, with 209 upregulated and 294 downregulated genes when compared to the IF group, whereas only 118 DEGs were detected in the PBMC group when compared to the IF group, which may reflect inferior fertility in the PBMC group mice and the fact that the PBMC group samples contained individuals destined for unsuccessful pregnancy (Fig. 3a, b).

Principal component analysis (PCA) showed that the PBMC group was distributed between the PBMC-hCG and IF groups (Fig. S3, Additional file 5) and overlapped with both the PBMC-hCG and IF groups. This finding suggests that the pregnant mice in the PBMC group had a gene expression similar to that in the PBMC-hCG group, and mice with unsuccessful pregnancies in this group had a gene expression similar to that in the IF group; therefore, we focused our subsequent analysis on gene expression changes in the PBMC-hCG group versus the IF group.

More than one-third of the DEGs in the IF group when compared to the control group overlapped with the DEGs in the PBMC-hCG group when compared to the IF group (Fig. 3c). In 84% of these overlapping genes (154 of 182 DEGs), the direction of gene expression changes in the PBMC-hCG and control groups relative to that in the IF group was consistent, suggesting that PBMC-hCG administration normalized the expression of many of the dysregulated genes in the IF group (Fig. 3d).

**Fig. 4:**
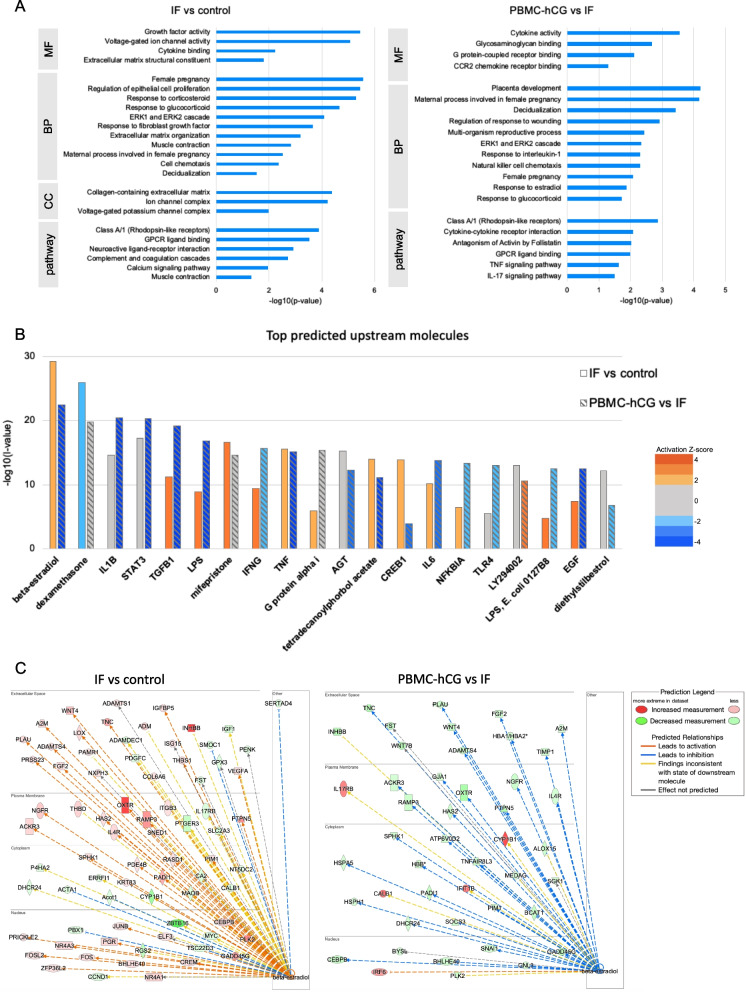
GO, pathway, and upstream molecule analyses of DEGs. Administration of hCG-treated PBMCs suppressed the expression of downstream genes of estrogen- and inflammation-related molecules and improved implantation-related genes. **a**, **b** Enrichment of functional annotations (**a**) and upstream regulators (**b**) in DEGs between PBMC-hCG and IF mouse uteri on the evening of 3.5 dpc. **a** GO, gene ontology; BP, biological process; CC, cellular component; MF, molecular function; pathway, Kyoto Encyclopedia of Genes (KEGG) and Reactome pathways. **b** Orange and blue bars indicate the activated and inhibited upstream molecules, respectively. Solid and striped bars indicate predicted upstream molecules for DEGs in IF versus control and PBMC-hCG versus IF, respectively. **c** Gene expression changes of β-estradiol responsive genes in each comparison

The results of the GO analysis using all DEGs for the IF versus control and PBMC-hCG versus IF groups are shown in Fig. 4a. Genes related to cytokine activity, pregnancy, decidualization, the ERK1/ERK2 cascade, response to glucocorticoids, and G protein-coupled receptor (ClassA/1) were significantly altered in common between the two group comparisons. The expression of a large proportion of the genes involved in these functions was ameliorated by PBMC-hCG administration (Fig. S4, Additional file 6). In addition, the expression of genes related to calcium signaling, muscle contraction, and epithelial cell proliferation was dysregulated in the IF group (Fig. S4, Additional file 6). By contrast, the expression of genes related to the estrogen and IL-1 response, and TNF and IL-17 signaling were significantly altered in the PBMC-hCG group (Fig. 4a).

Upstream molecular analysis of DEGs showed that genes downstream of estradiol were activated in the IF group when compared to the control group and were suppressed in the PBMC-hCG group when compared to the IF (Fig. 4b, c). Furthermore, the top upstream molecules of DEGs common to both IF versus control and PBMC-hCG versus IF included many inflammation-related molecules such as LPS, IFNγ, IL-6, NFκBIA, and tetradecanoylphorbol acetate; an immune cell stimulating agent (Fig. 4b). These results suggested that the estrogen-activated and inflammation-related genes in the endometrium, which were activated in the IF model, were suppressed by PBMC-hCG administration.

### Transcriptomic changes of estrogen-responsive genes and embryo implantation-associated genes by PBMC administration

**Fig. 5:**
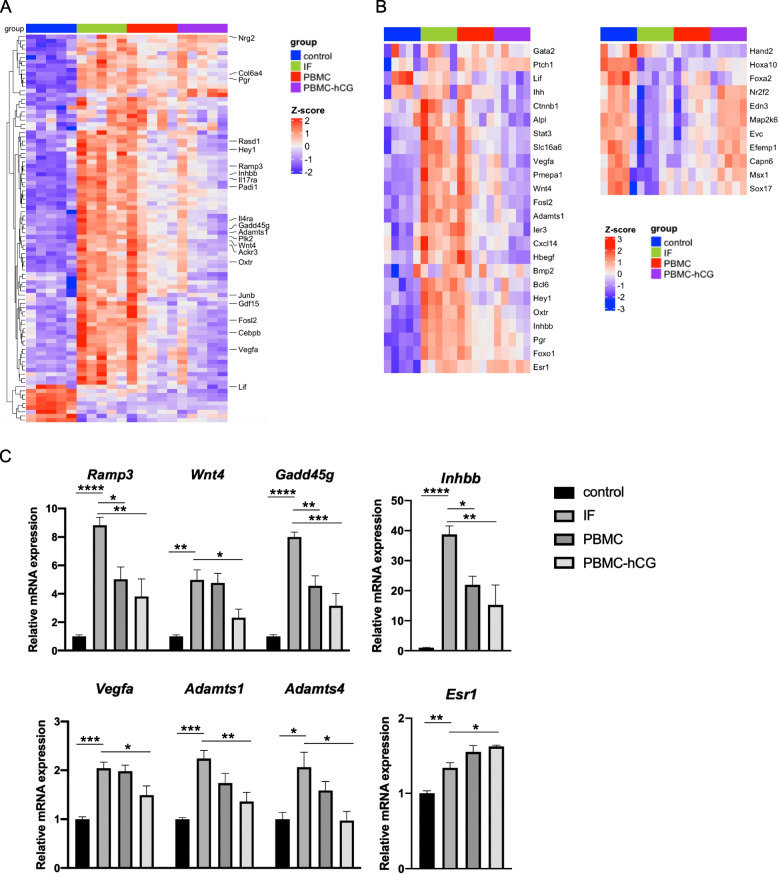
Expression of estrogen-responsive genes and uterine function-related genes. Abnormal expression of estrogen-responsive genes and genes involved in decidualization and endometrial epithelial polarity was rescued by the administration of PBMCs, especially hCG-treated PBMCs. **a** Heatmap of the estrogen-responsive genes in the mouse uterus of DEGs in IF versus control mice. **b** Heatmap of embryo implantation and uterine function-associated genes. **c** Quantification of estrogen-responsive genes and estrogen receptor α gene in 3.5 dpc uteri of each group. * *p* < 0.05, ** *p* < 0.01, *** *p* < 0.005, **** *p* < 0.0001

We examined the gene expression changes in each group for estrogen-responsive genes in the mouse uterus (extracted from GSE133158) and implantation-related genes, as shown in the heatmap (Fig. 5a, b). The majority of estrogen-responsive genes were overexpressed in the IF group and downregulated in the PBMC and PBMC-hCG groups, and this change was more pronounced in the PBMC-hCG group (Fig. 5a). With regard to known mouse and human implantation-related genes, the dysregulated expression of genes involved in decidualization (*Wnt4*, *Stat3*, and *Fosl2*) and the loss of polarity of the luminal epithelium during embryo implantation (*Msx1* and *Stat3*) were ameliorated in the PBMC-hCG group. Both *Msx1* and *Stat3* were down- and upregulated, respectively, following the estrogen surge at day 3.5 just prior to embryo implantation. However, these fluctuations in expression were excessive in the IF model and mitigated by PBMC administration. The expression of *Capn6*, *Map2k6*, and *Impa2*, which are included in the endometrial receptivity array (ERA) gene list [27] and are known to be dysregulated by controlled ovarian stimulation, was also ameliorated in the PBMC-hCG group. However, the dysregulated expression of progesterone receptor target genes (*Ihh*, *Gata2*, and *Hand2*) and *Lif*, which are important for embryo implantation, was not corrected by PBMC administration (Fig. 5b).

qRT-PCR also confirmed that the expression of estrogen-activated genes (*Inhbb*, *Ramp3*, *Wnt4*, *Gadd45g*, *Vegfa*, *Adamts1*, and *Adamts4*) was rescued in PBMC-treated mice, especially in the PBMC-hCG group (Fig. 5c). To investigate the causes of these changes in estrogen-responsive genes, we examined the blood estrogen levels and estrogen receptor expression in the endometrium on day 3.5. Estrogen receptor α was upregulated in both the IF and PBMC-hCG groups and was not suppressed by PBMC administration (Fig. 5c). Plasma estradiol levels did not differ significantly between the IF versus control and IF versus PBMC/PBMC-hCG groups (Fig. S5, Additional file 7).

### Changes in inflammation-related genes by PBMC administration

**Fig. 6:**
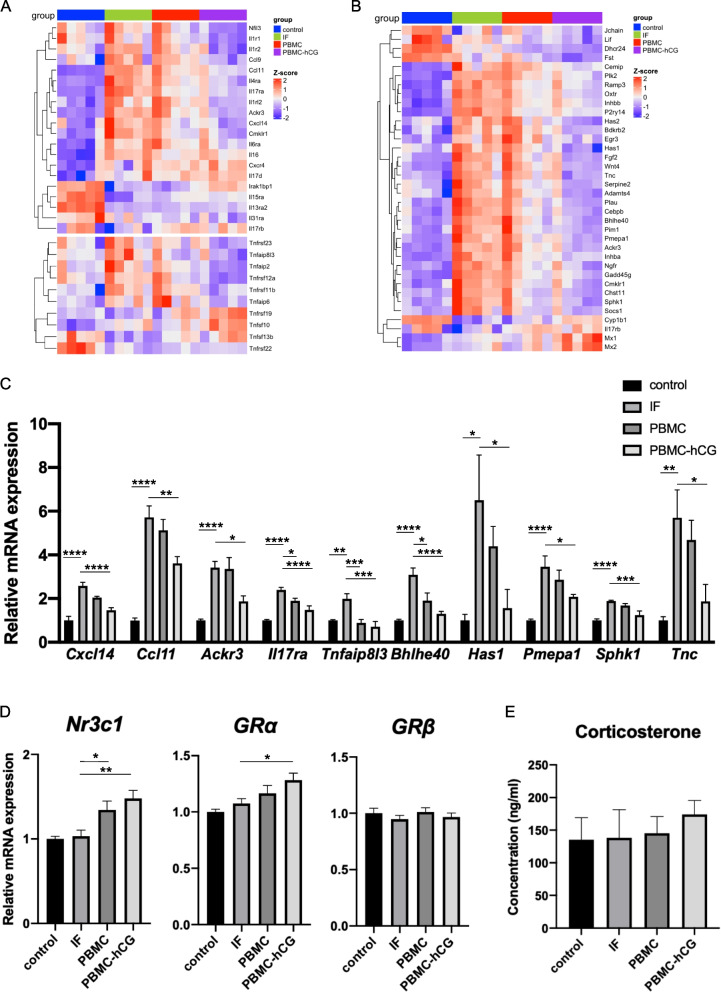
Expression of inflammation-related genes. PBMC administration suppressed genes associated with interleukin and TNF signaling and downstream genes of inflammation-related molecules, with increased expression of glucocorticoid receptor-α. **a** Heatmap of immune-related genes, including cytokine or chemokine ligands, receptors, and TNF signaling proteins, in DEGs between IF versus control or PBMC-hCG versus IF. **b** Heatmap of downstream genes of inflammatory molecules (TNF, IL-6, LPS, NFκBIA, and RELA). **c** Normalized gene expression levels from the RNA-seq data of representative inflammation-related genes in each group. * *p* < 0.05, ** *p* < 0.01, *** *p* < 0.005, **** *p* < 0.001. **d** Quantification of glucocorticoid receptor mRNA in 3.5 dpc uteri of each group. * *p* < 0.05, ** *p* < 0.01. **e** Plasma corticosterone concentration in the evening at 3.5 dpc (*n* = 4–5 per each group). No significant difference was observed by one-way ANOVA test

Because many of the cytokine genes were expressed at very low levels in the mouse uterus and comparative analysis of their expression changes was difficult, we examined the gene expression of receptors and downstream signaling molecules of cytokines (interleukins, chemokines, and TNF). Dysregulated genes related to IL-1, IL-6, IL-17, and TNF signaling were ameliorated in the PBMC-hCG group (Fig. 6a). In addition, many downstream genes of inflammation-related molecules included in the top 20 upstream factors of the DEGs were activated in the IF group and suppressed in the PBMC-hCG group (Fig. 6b). PCR validation also showed that IL17, TNF, chemokine signaling-related molecules, and genes downstream of TNF or LPS were downregulated in the PBMC-hCG group (Fig. 6c). We also evaluated the GR gene expression, which is thought to be involved in the regulation of inflammatory responses, and found that the GR gene (*Nr3c1*) RNA level was upregulated in the PBMC and PBMC-hCG groups compared to that in the IF group (Fig. 6d). GRα, which is involved in the suppression of inflammatory responses, was significantly upregulated in the PBMC-hCG group, while GRβ, which inhibits GRα, was not altered in the PBMC and PBMC-hCG groups (Fig. 6d). Plasma corticosterone levels on the evening of day 3.5 did not differ between the IF and control groups, and were not altered by PBMC/PBMC-hCG administration (Fig. 6e). Additionally, the RNA-seq results revealed a strong correlation between the expression of GR (*Nr3cl*) and implantation-related genes *Msx1* and *Oxtr* in each sample (IF, PBMC, and PBMC-hCG groups; Fig. S6, Additional file 8).

## Discussion

RIF is a major challenge in reproductive medicine, often linked to dysregulated endometrial receptivity and inflammatory responses. Our analysis revealed that intrauterine PBMC administration decreases the expression of estrogen-responsive and inflammation-related genes in the endometrium during the implantation phase. We also confirmed for the first time that hCG enhances the therapeutic effect of PBMC administration in vivo. In this study, we demonstrated that intrauterine administration of PBMC not only increased the number of embryo implantations, as previously reported [15, 17], but also improved the rate of normal embryo implantation in a mouse model of implantation failure. Studies in mice and in vitro study using human cells [18] have suggested that PBMCs improve endometrial receptivity; however, for ethical reasons, collecting endometrial samples from patients with RIF at the implantation stage after PBMC administration is difficult. Therefore, we performed RNA-seq analysis of the uterus immediately before implantation in this mouse model to investigate the effects of PBMCs on the implantation phase and the underlying mechanism. We further examined the effect of hCG in this study, since hCG-treated PBMC administration is used in many clinical PBMC therapies as immunotherapy with a high level of evidence [28]; however, the efficacy of PBMC therapy with or without hCG has not been verified. The results showed that the number of normal-sized implantation sites, which are considered normallydeveloped, was significantly higher in mice treated with hCG-treated PBMCs than in those treated with PBMCs without hCG. The pregnancy rate and the number of embryo implantations also tended to be higher in mice treated with hCG-treated PBMCs. RNA-seq analysis showed that a large proportion of dysregulated genes in the mouse implantation failure model were rescued by PBMC administration, especially with hCG-treated PBMCs, and the degree of improvement was consistent with the pregnancy outcomes of each group.

Among the various factors required for successful embryo implantation into the endometrium, steroid hormones, especially progesterone and estrogen, are important. Excessive estrogen just before the implantation phase increased uterine fluid levels via altered aquaporin expression, resulting in implantation failure in mice [29]. Conversely, progesterone suppresses estrogenic effects, thereby creating the endometrial environment necessary for embryo implantation during the implantation phase [30]. A meta-analysis on the transcriptome analysis of the endometrium of patients with RIF identified ESR1 as a common upstream factor of dysregulated genes in RIF [31]. The estrogen/progesterone signaling balance of the endometrium is crucial for embryo implantation and an imbalance in estrogen action could be a cause of implantation failure. Examination of implantation-related genes revealed that the abnormal expression of *Msx1* and *Oxtr*, which are regulated by estrogen [32, 33], was rescued by PBMC-hCG treatment. *Msx1/2* suppresses the activity of estrogen-activated genes in the epithelium via *Wnt* and β-catenin signaling, regulates the polarity of epithelial cells during implantation, and is known to be involved in the expression of *Bmp2* required for decidualization [34, 35]. Oxytocin signaling enhances uterine contractions, and blocking oxytocin receptor (OXTR) using antagonists has been shown to improve IVF outcomes [36]. In our study, the upregulation of genes related to uterine contractions, including *Oxtr*, was normalized by PBMC-hCG. Given these findings and the result that estrogen-responsive genes were suppressed by PBMC-hCG, we speculate that PBMC-hCG treatment favorably alters the endometrium and intrauterine environment for embryo implantation by rectifying the aberrant expression of implantation-related genes through improving the balance of estrogenic effects.

In addition to the estrogen/progesterone balance, glucocorticoid signaling is important for establishing and maintaining normal pregnancy. Uterine GR knockout mice display reduced embryo implantation and impaired decidualization [37], and a gene expression analysis comparing the endometrium of RIF and controls reported that immune-related genes are altered in RIF [38–40]. PBMC administration may also improve pregnancy outcomes by modulating glucocorticoid signaling and the inflammatory response in the endometrium, similar to immunosuppressive therapies, thereby improving endometrial receptivity.

PBMC administration suppressed estrogen-responsive genes in the endometrium but did not reduce estrogen receptor (ER) expression or blood levels of estradiol, the ER ligand. In addition, it did not lead to certain directional changes in the expression of progesterone downstream genes, nor did it ameliorate the decrease in *Hand2* expression, which is involved in suppressing estrogen signaling downstream of progesterone, suggesting that PBMC administration does not suppress estrogen signaling via the regulation of progesterone signaling. By contrast, the expression of GRα, the receptor mediating glucocorticoid action, in the endometrium was significantly upregulated in the PBMC-treated group. In addition, the RNA-seq results showed a strong correlation between GR (*Nr3cl*) and *Msx1* and *Oxtr* expression, suggesting that an altered GR expression improves the expression of these implantation-related genes.

**Fig. 7:**
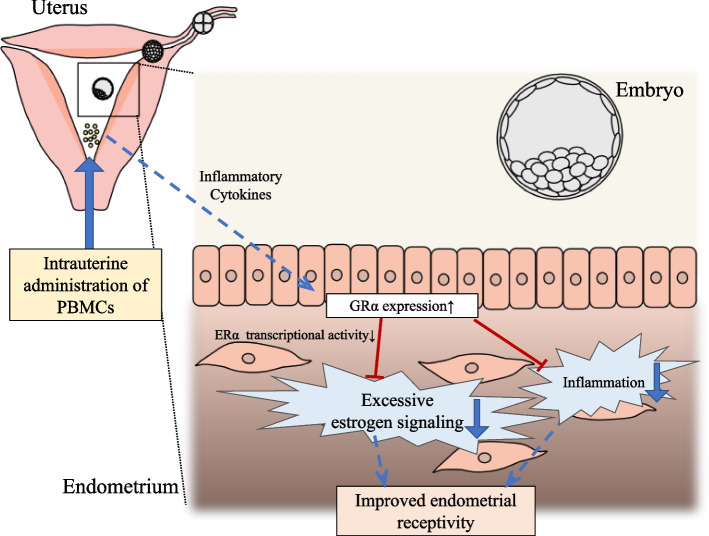
Schematic diagram illustrating the presumed mechanisms of improvement in endometrial receptivity by PBMC treatment. Intrauterine administration of PBMCs improves endometrial receptivity, possibly by inflammatory cytokine production and the subsequent modulation of glucocorticoid receptor expression. This downstream effect suppresses excessive estrogen and inflammatory signaling, thereby improving the endometrial receptivity

Both ER and GR affect each other's transcriptional activity through common DNA-binding regions and transcriptional regulators, and several genes are regulated by both estrogen and glucocorticoids in various tissues [41–43]. Some gene clusters are antagonistically regulated by estrogen and glucocorticoids in human endometrial epithelial cell lines and in mouse and rat uteri [44–46], probably through ER- and GR-mediated antagonism [43, 46]. Inflammatory cytokines such as IL-1β and TNF-α have been reported to increase GR expression in endometrial stromal cells [47, 48], and inflammatory stimulation using LPS increased GRα expression in multiple cell types [49]. Considering these findings and our results, PBMC administration may potentially increase GR expression and enhance GR signaling via inflammatory cytokines that could be locally produced by PBMC themselves. This might lead to the suppression of the ER transcriptional activity through the interaction between GR and ER and the restoration of dysregulated genes that are activated by estrogen (Fig. 7). However, further studies are required to confirm this proposed mechanism.

In this study, mice treated with PBMC-hCG exhibited outcomes more closely resembling those of normal pregnant mice compared to mice treated with PBMC, both in terms of pregnancy and endometrial gene expression. hCG acts on the endometrium to regulate LIF and VEGF expression, induces immune tolerance by enhancing tolerogenic dendritic cells, regulatory T cells, and regulatory B cells, and enhances the interaction between the embryo and endometrium [50–54]. Conversely, the supplementation of hCG in PBMC culture increases the production of inflammatory cytokines such as IL-1β, TNFα, and IL-8 by PBMCs [7, 15, 55]. Thus, the enhanced effect of PBMC therapy on the endometrium with the addition of hCG is presumably attributed not only to the direct regulatory effect of hCG on the endometrium but also to the increased action of PBMC-derived cytokines, which are amplified by hCG. However, further investigations are required to clarify the factors that contribute to this enhanced therapeutic effect.

One limitation of this study is that we could not examine the effects of PBMC administration on the human endometrium. However, collecting endometrial samples from patients with RIF during the implantation stage is ethically challenging. Additionally, the 2D cultures of human implantation models cannot fully mimic the intrauterine environment. The 3D implantation model is a promising tool for representing in vivo human endometrial function, and verification of the action of PBMCs on embryo implantation using this model is expected in future studies.

## Conclusions

In conclusion, we demonstrated for the first time that intrauterine PBMC administration, especially after hCG-added culture, improves embryo implantation by normalizing aberrant gene expression in the endometrium during the implantation phase by suppressing excessive estrogen-regulated gene activity and inflammation. Our data suggest that PBMC therapy is effective in correcting impaired embryo receptivity due to hormonal imbalance in the endometrium. However, further examination in humans is warranted.

## Supplementary Information

Additional file 1: Supplemental Tables S1 and S2. Antibodies used in immunostaining and primers utilized for real-time q-PCR.

Additional file 2. Material and Methods (detailed).

Additional file 3: Fig. S1. Effect of hCG-treated PBMC administration on uterine morphology at the peri-implantation stage. Immunohistochemical analysis of the morphology and ki-67 expression in each group of uteri on the morning and evening of 3.5 dpc. Samples were not collected from the IF group on the morning of 3.5 dpc, as RU486 was administered to this group on the morning of 3.5 dpc. The scale bar represents 1 mm.

Additional file 4: Fig. S2. HE stained histological images of uteri in the PBMC and IF group at 7.5 dpc. Uteri of the PBMC group mice showed different histology depending on the size of the implantation site (normal, intermediate, or small) (a). Uteri of the IF group mice showed no fetus or changes of surrounding tissue (b). The scale bar represents 500 µm.

Additional file 5: Fig. S3. Principal component analysis (PCA) of RNA-seq data. The PCA of all 20 samples showed that the PBMC-hCG group had relatively similar gene expression to that of the control group in PC2. The distribution of the PBMC group was intermediate between that of the IF and PBMC-hCG groups, consistent with pregnancy outcomes.

Additional file 6: Fig. S4. Relative expression of genes in enriched GO terms. The heatmap shows the relative expression of DEGs in GO terms related to female reproduction and GPCR, including chemokine and growth factor receptors. Ca signaling = Calcium ion transport and calcium signaling pathway.

Additional file 7: Fig. S5. Plasma level of β-estradiol in each group on the evening of 3.5 dpc. HCG-treated PBMC administration did not affect plasma E2 level at peri-implantation stage.

Additional file 8: Fig. S6. Correlation between the expression levels of GR (*Nr3c1*) and *Msx1* and *Oxtr.* The figure shows the correlation of normalized log2 values for *Nr3c1* (glucocorticoid receptor gene), *Msx1*, and *Oxtr* in each sample within the IF, PBMC, and PBMC-hCG groups. Spearman's rank correlation test was performed to determine the strength of the correlation.

## Data Availability

RNA Sequence data that support the findings of this study have been deposited in the Sequence Read Archive with the BioProject accession number PRJNA1171016.

## Abbreviations

ANOVA: One-way analysis of variance
DEG: Differentially expressed genes
GO: Gene ontology
ER: Estrogen receptor
ERA: Endometrial receptivity array
GR: Glucocorticoid receptor
HE: Hematoxylin–eosin
IS: Implantation site
IVF: In vitro fertilization
KEGG: Kyoto Encyclopedia of Genes and Genomes
OXTR: Oxytocin receptor
PBMC: Peripheral blood mononuclear cells
PCA: Principal component analysis
qPCR: Quantitative PCR
RIF: Recurrent implantation failure
TMM: Trimmed mean of M-value
