# Supplementary material for: Intrauterine administration of peripheral blood mononuclear cells helps manage recurrent implantation failure by normalizing dysregulated gene expression including estrogen-responsive genes in mice
Source: Cell Commun Signal. 2024 Dec 5;22:587. doi: 10.1186/s12964-024-01904-3 (PMC11619271; doi:10.1186/s12964-024-01904-3)
Supplement: Supplementary file 1 — Additional file 1: Supplemental Tables S1 and S2. Antibodies used in immunostaining and primers utilized for real-time q-PCR. [file 12964_2024_1904_MOESM1_ESM.docx]

**Additional file 1: Supplemental Tables**

**Table S1. Antibodies used in immunostaining**

| Antibody | Type | Manufacturer | Catalog No. | Dilution for IHC |
| --- | --- | --- | --- | --- |
| Ki-67 | Rat monoclonal | eBioscience | 14-5698-82 | 1:200 |

**Table S2. Primers utilized for real-time q-PCR**

| Gene | Forward | Reverse |
| --- | --- | --- |
| *Adamts1* | AACGGTTCCACATGCAAGAAGATG | TGGTGGCTCCAGCAGGAATTG |
| *Adamts4* | CTGCAGTGCCCGATTCATCAC | TGGCGGTCAGCGTCATAGTC |
| *Esr1* | CTGCGCAAGTGTTACGAAGTG | TCTCTGACGCTTGTGCTTCAAC |
| *Gadd45g* | CCAGGATACAGTTCCGGAAAGC | CACATTGTCAGGGTCCACATTCAG |
| *Inhbb* | AGATCATCAGCTTTGCAGAGACA | CTGGCCTGCACCACGAATAG |
| *Ramp3* | AGAAGGTGGCTGTCTGGAAGTG | TCTCCATCTCGGTGCAGTTAGTG |
| *Vegfa* | CAGCAGATGTGAATGCAGACCAA | GGTGACATGGTTAATCGGTCTTTCC |
| *Wnt4* | GGCACTCATGAATCTTCACAACAAC | GCACGCCAGCACGTCTTTAC |
| *Ackr3* | CCGTCAGGAAGGCAAACCA | CCAGGCTCTGCATAGTCAAACA |
| *Bhlhe40* | AGGATCTCCTACCCGAACATCTC | TCAATGCTTTCACGTGCTTCAAC |
| *Ccl11* | CCAGGCTCTGCATAGTCAAACA | TGTCAAGAGAGGAGGTTGTTTATGG |
| *Cxcl14* | CCTAAGCTGCAGAGCACCAAAC | CCATGATCGTCCACCCTATTCTTC |
| *Has1* | ACGTGAGGTCATGTACACAGCTTTC | AGTGCCATGGGGTCTAGTCTTG |
| *Il17ra* | ACCCAGCACGGAGAATTAGTC | AGCCGCTCATTGGTGTTCAG |
| *Pmepa1* | AACTGCCAGCGCTCTTTGTTC | CGGGCTGACAGCTTGTAGTG |
| *Sphk1* | CAGCCCAGTGCTTCAGTTGTC | GCTCCTGCGTTCAGCTTCTTATC |
| *Tnc* | TGGTCCATCTGGTCTTCTGATAGC | CCAGACACTGTGCGTGTAACTTC |
| *Tnfaip8l3* | CTCCAGACTCTTTGCAGCCCATAC | AAGGGCGAGATTCTTGGAACTAAAC |
| *Nr3c1* | GGTCTGGAGAGGACAACCTGAC | AGCTGGACGGAGGAGAACTCAC |
| *Nr3c1(GRα)* | AAAGAGCTAGGAAAAGCCATTGTC | CTGTCTTTGGGCTTTTGAGATAGG |
| *Nr3c1(GRβ)* | AAAGAGCTAGGAAAAGCCATTGTC | TCAGCTAACATCTCTGGGAATTCA |
| *Rplp0* | CCCTGCACTCTCGCTTTCTG | GACGCGCTTGTACCCATTGA |
| *Gapdh* | GAGTGTTTCCTCGTCCCGTAGAC | TTGATGGCAATCTCCACTTTGC |
