# Supplementary material for: Intrauterine administration of peripheral blood mononuclear cells helps manage recurrent implantation failure by normalizing dysregulated gene expression including estrogen-responsive genes in mice
Source: Cell Commun Signal. 2024 Dec 5;22:587. doi: 10.1186/s12964-024-01904-3 (PMC11619271; doi:10.1186/s12964-024-01904-3)
Supplement: Supplementary file 2 — Additional file 2. Material and Methods (detailed). [file 12964_2024_1904_MOESM2_ESM.docx]

**Additional file 2: Material and Methods (detailed)**

**Immunohistochemistry**

Frozen sections of the uterine horns were prepared as previously described, with slight modifications. Briefly, the fresh tissue samples of uterine horns were fixed in 4% paraformaldehyde (Nacalai Tesque, Tokyo, Japan) at 4°C for 1 h, transferred into 30% sucrose, embedded in OCT compound (Miles, Elkhart, USA), snap-frozen in liquid nitrogen, and stored at -80°C until use. The embedded tissue samples were sectioned into 8-μm thick sections on glass slides and fixed with acetone (Nacalai Tesque) at -20°C for 5 min.

Antigen retrieval was performed by incubating sections for 20 min in 10 mM citrate buffer (PH 6.0) at 90℃. Then, sections were blocked with 5% goat serum + 0.2% triton X-100 + 0.02% AZ in PBS for 30 min and incubated with primary antibody overnight at 4℃. The sections were treated with 3% H_2_O_2_ in PBS for 10 min to block peroxidase activity. They were then incubated with a biotinylated goat anti-rat secondary antibody (1:1000, Jackson ImmunoResearch, USA) for 30 min, followed by incubation with a streptavidin-peroxidase complex solution (Nichirei Biosciences, Tokyo, Japan) for 30 min. The sections were stained with 3,3′-diaminobenzidine (DAB) and lightly counterstained with hematoxylin. Images of the IHC staining were captured using an optical microscope (IX71; Olympus, Tokyo, Japan).

**Transcriptome data analysis**

GO term and KEGG/Reactome pathway enrichment analyses was performed on DEGs in two comparisons (IF versus control and PBMC-hCG versus IF groups) using gProfiler with the term size set to 4–500 and a corrected p-value threshold of 0.05. Identification of upstream regulators of DEGs in the same two comparisons was performed using the Ingenuity Pathway Analysis software (Qiagen). Upstream molecules were defined as significant by default cut-off values (z-score of ≥ +2 or ≤ -2 predicted activation or inhibition, respectively), and chemical toxicants and complexes were excluded. The top20 molecules were depicted on the graph in order of decreasing p-values of overlap (minimum value in two comparisons), which indicates the significance of overlap between genes in the DEGs and known targets regulated by upstream molecules. Spearman's rank correlation test was performed on the normalized expression values of GR and implantation-related genes for the three groups (IF, PBMC, and PBMC-hCG) to investigate the effect of PBMC administration in the IF model. DEGs extraction, principal component analysis, correlation analysis, and data visualization were performed using R version 4.0.5 (R Core Team 2021, Vienna, Austria).
