# Supplementary figures and images for: Intrauterine administration of peripheral blood mononuclear cells helps manage recurrent implantation failure by normalizing dysregulated gene expression including estrogen-responsive genes in mice
Source: Cell Commun Signal. 2024 Dec 5;22:587. doi: 10.1186/s12964-024-01904-3 (PMC11619271; doi:10.1186/s12964-024-01904-3)

control

IF

PBMC-hCG

3.5 dpc 9:00

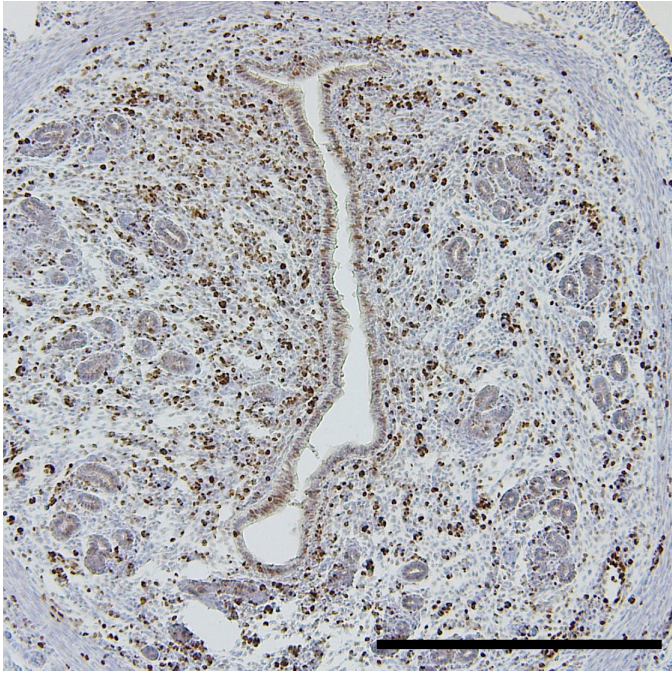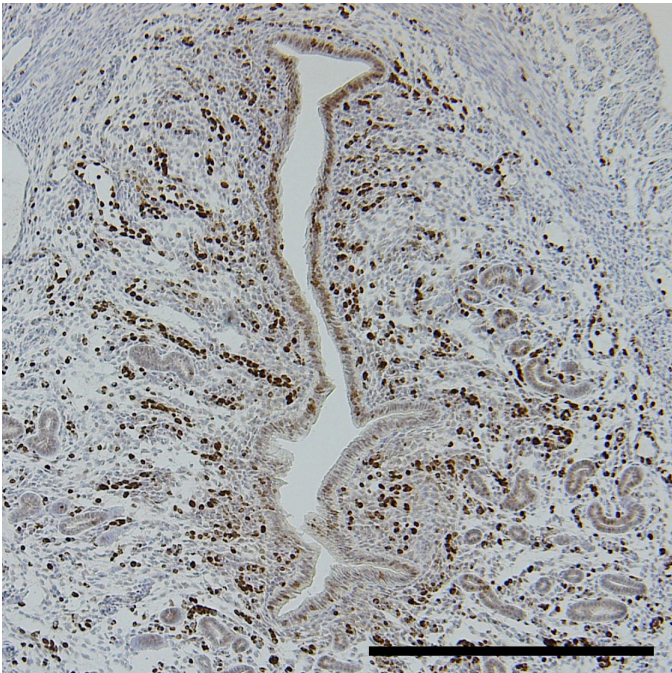

3.5 dpc 18:00

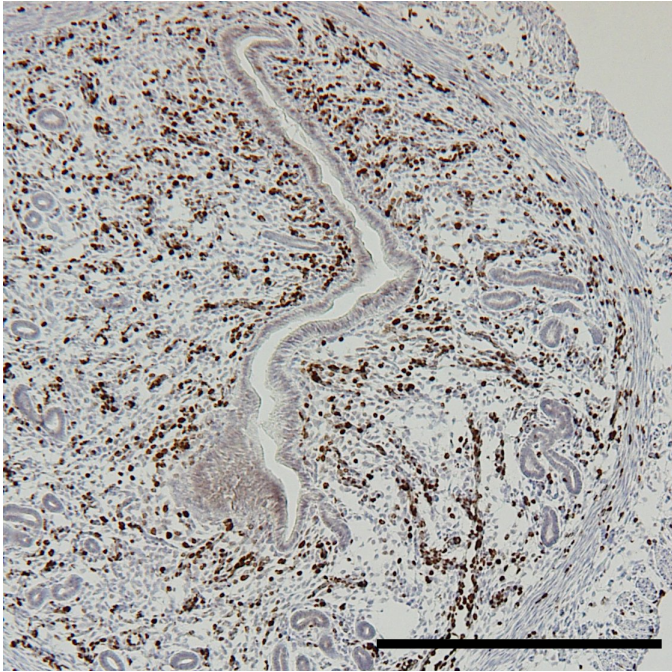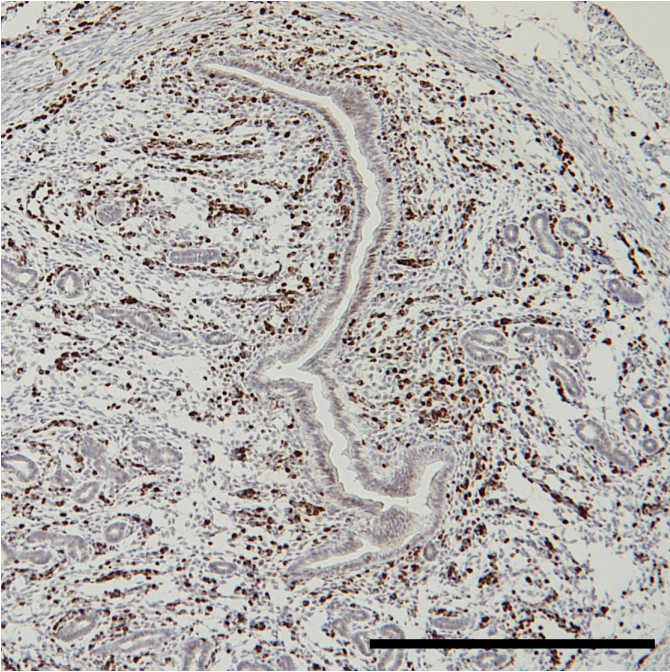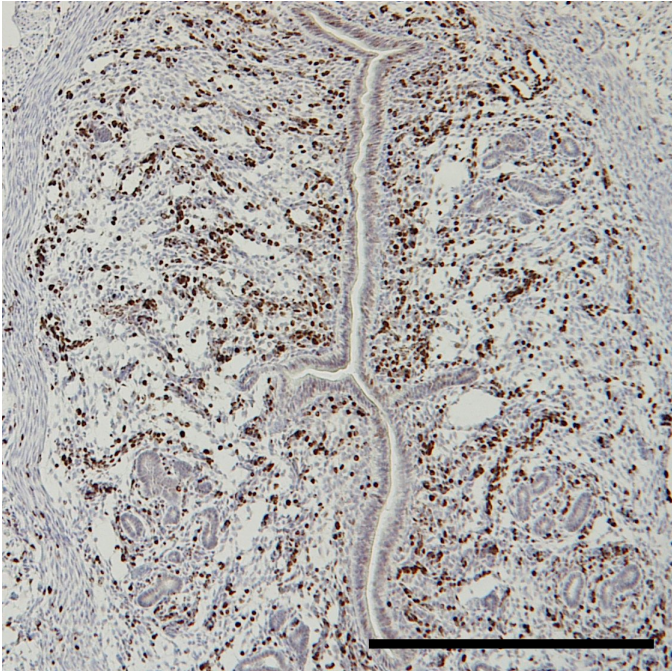

Supplement: Supplementary file 3 — Additional file 3: Fig. S1. Effect of hCG-treated PBMC administration on uterine morphology at the peri-implantation stage. Immunohistochemical analysis of the morphology and ki-67 expression in each group of uteri on the morning and evening of 3.5 dpc. Samples were not collected from the IF group on the morning of 3.5 dpc, as RU486 was administered to this group on the morning of 3.5 dpc. The scale bar represents 1 mm. [file 12964_2024_1904_MOESM3_ESM.pdf]

A

Normal-sized IS

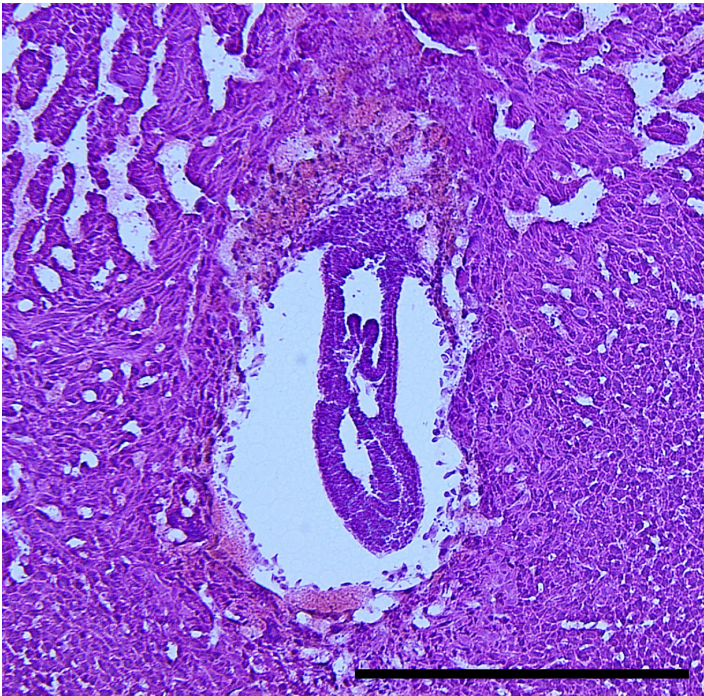

Intermediate-sized IS

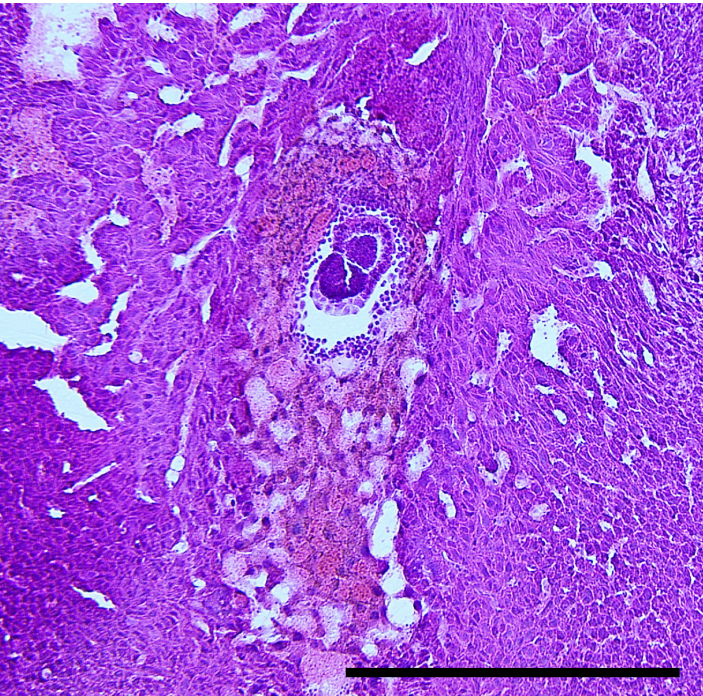

Small IS

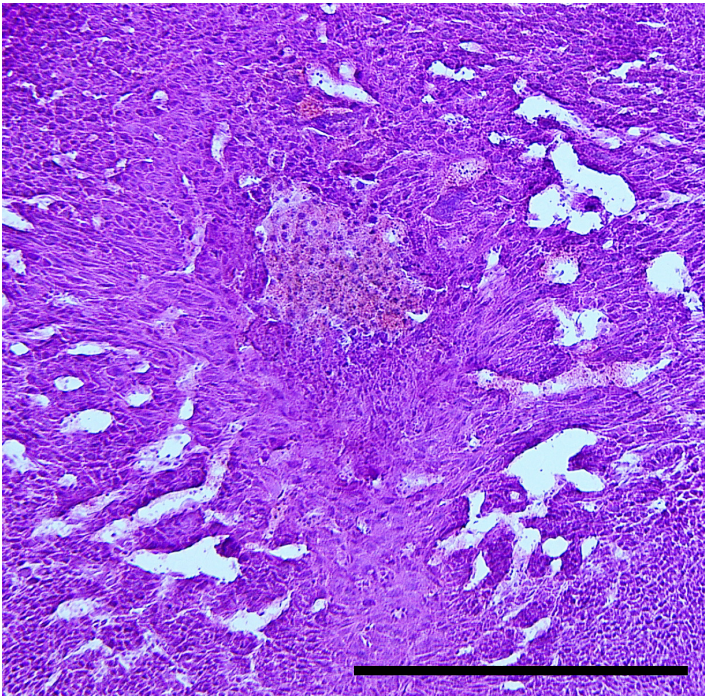

B

IF

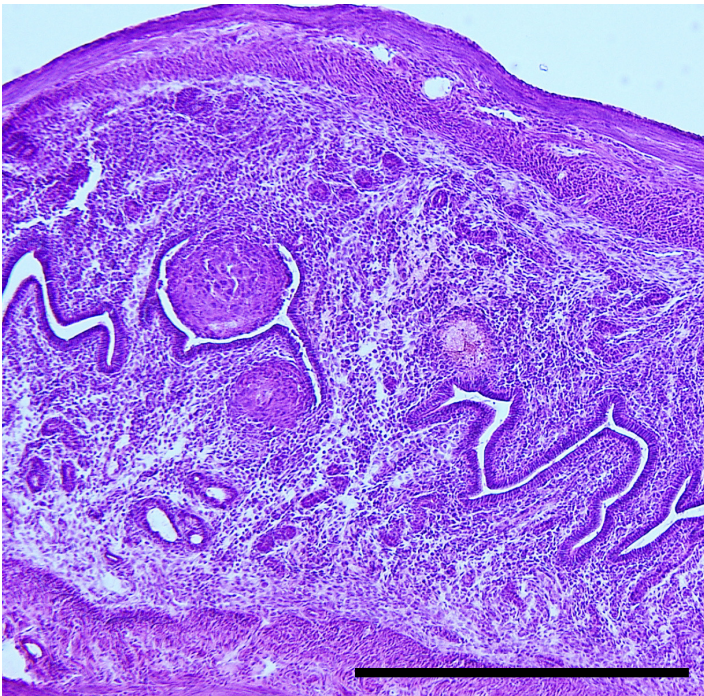

Supplement: Supplementary file 4 — Additional file 4: Fig. S2. HE stained histological images of uteri in the PBMC and IF group at 7.5 dpc. Uteri of the PBMC group mice showed different histology depending on the size of the implantation site (normal, intermediate, or small) (a). Uteri of the IF group mice showed no fetus or changes of surrounding tissue (b). The scale bar represents 500 µm. [file 12964_2024_1904_MOESM4_ESM.pdf]

# PCA

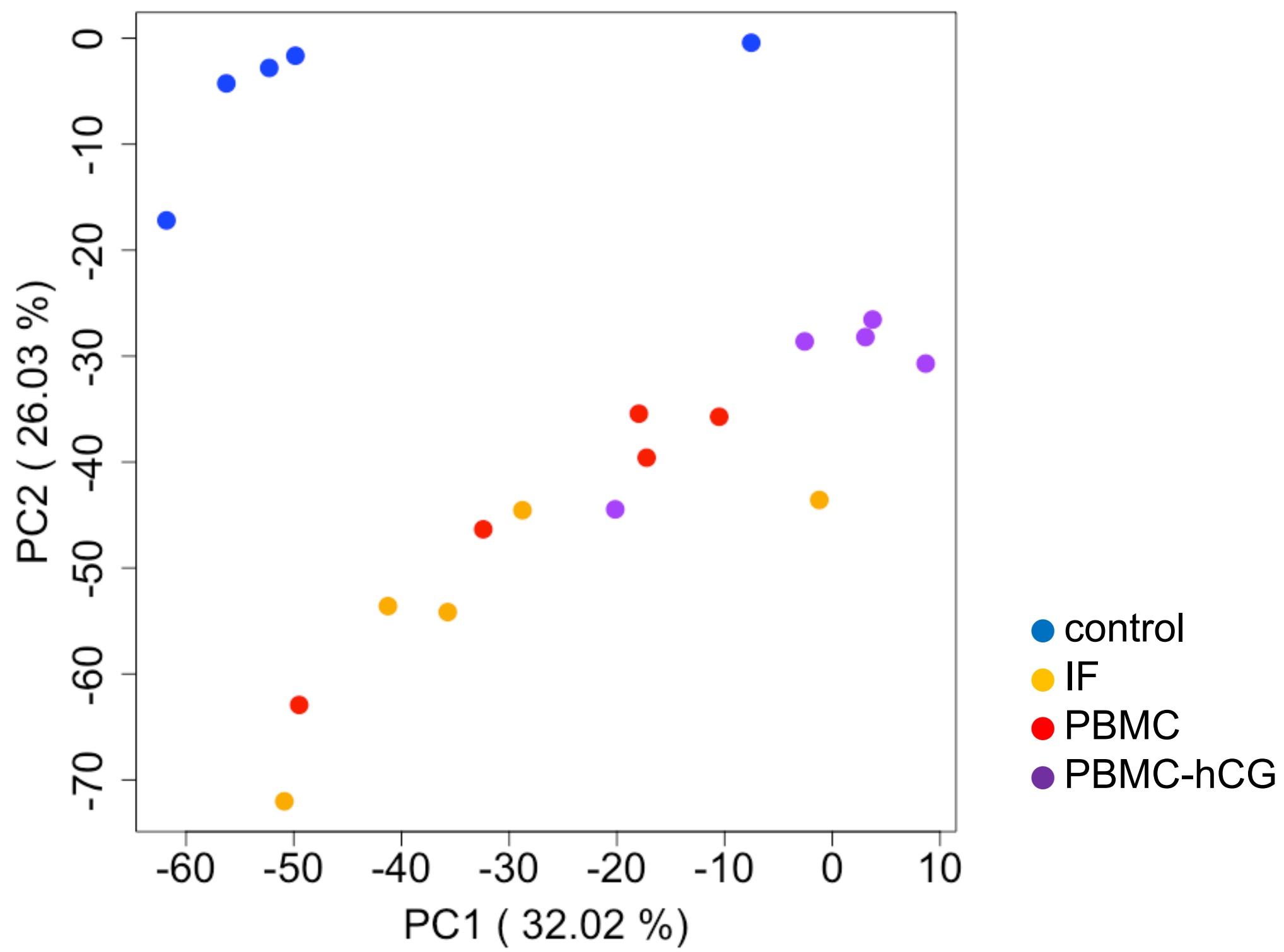

Supplement: Supplementary file 5 — Additional file 5: Fig. S3. Principal component analysis (PCA) of RNA-seq data. The PCA of all 20 samples showed that the PBMC-hCG group had relatively similar gene expression to that of the control group in PC2. The distribution of the PBMC group was intermediate between that of the IF and PBMC-hCG groups, consistent with pregnancy outcomes. [file 12964_2024_1904_MOESM5_ESM.pdf]

# $\beta$ -Estradiol

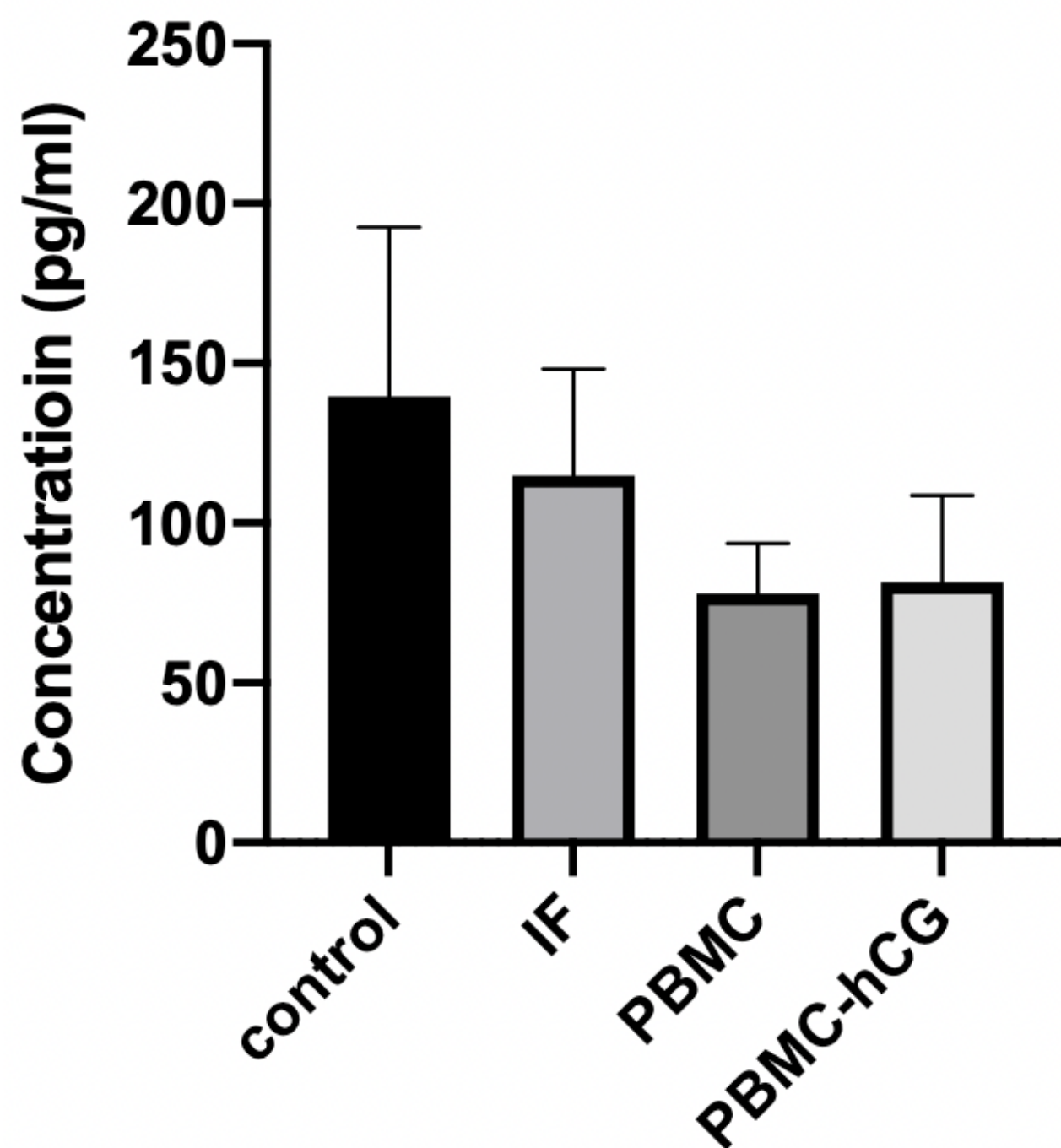

Supplement: Supplementary file 7 — Additional file 7: Fig. S5. Plasma level of β-estradiol in each group on the evening of 3.5 dpc. HCG-treated PBMC administration did not affect plasma E2 level at peri-implantation stage. [file 12964_2024_1904_MOESM7_ESM.pdf]

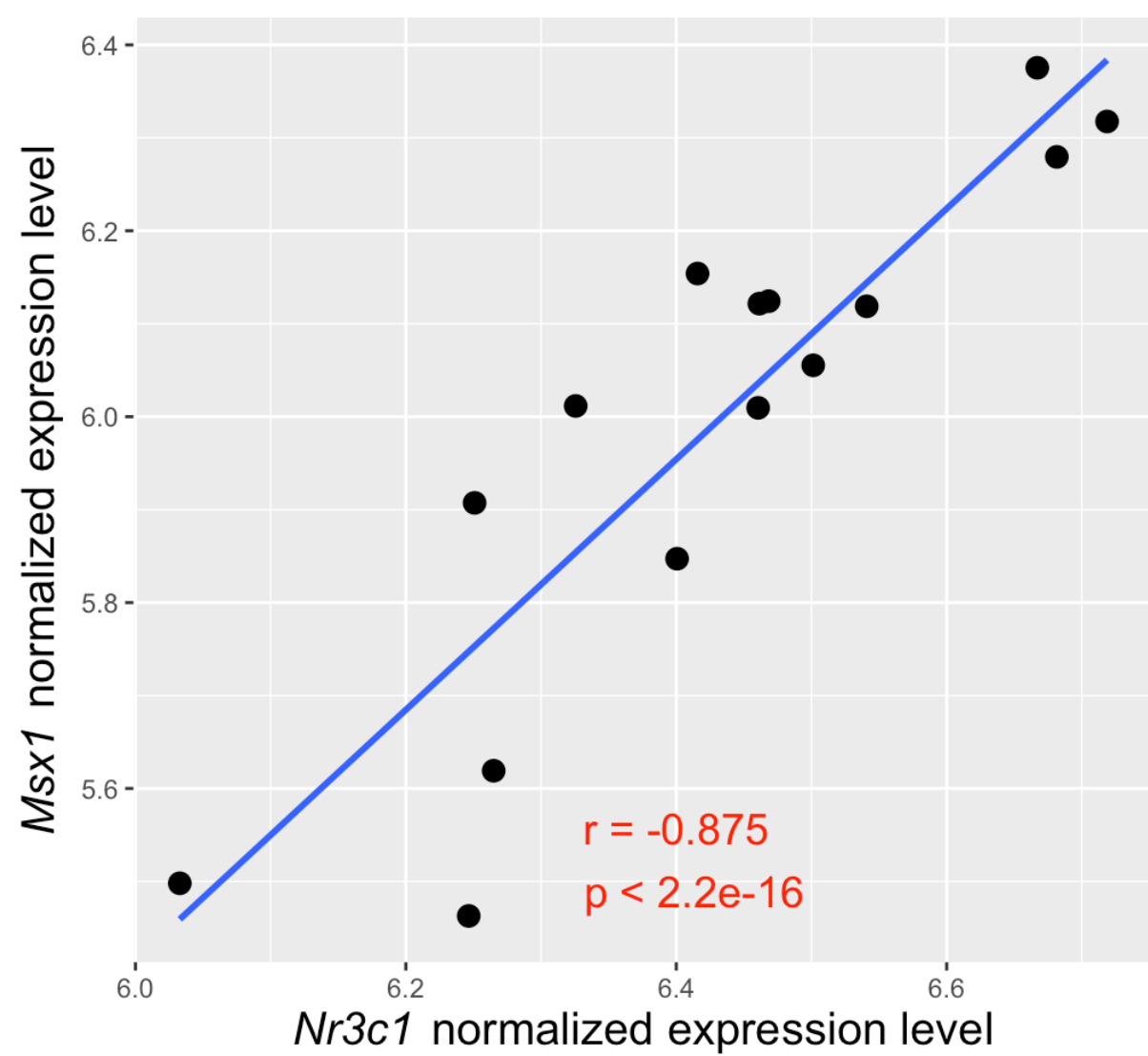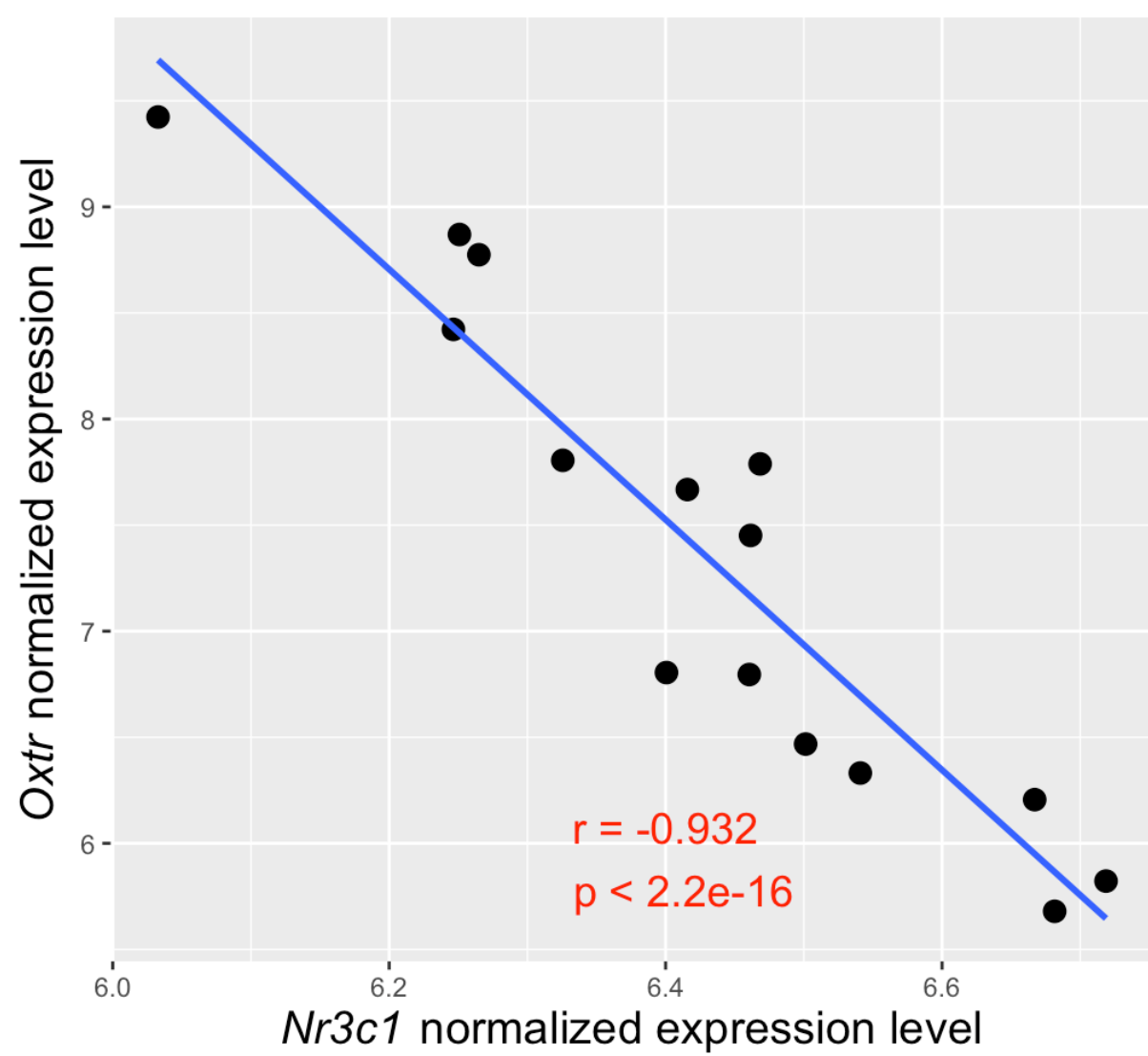

Supplement: Supplementary file 8 — Additional file 8: Fig. S6. Correlation between the expression levels of GR (Nr3c1) and Msx1 and Oxtr. The figure shows the correlation of normalized log2 values for Nr3c1 (glucocorticoid receptor gene), Msx1, and Oxtr in each sample within the IF, PBMC, and PBMC-hCG groups. Spearman's rank correlation test was performed to determine the strength of the correlation. [file 12964_2024_1904_MOESM8_ESM.pdf]
