# Supplementary material for: Intrauterine administration of peripheral blood mononuclear cells helps manage recurrent implantation failure by normalizing dysregulated gene expression including estrogen-responsive genes in mice
Source: Cell Commun Signal. 2024 Dec 5;22:587. doi: 10.1186/s12964-024-01904-3 (PMC11619271; doi:10.1186/s12964-024-01904-3)
Supplement: Supplementary file 6 — Additional file 6: Fig. S4. Relative expression of genes in enriched GO terms. The heatmap shows the relative expression of DEGs in GO terms related to female reproduction and GPCR, including chemokine and growth factor receptors. Ca signaling = Calcium ion transport and calcium signaling pathway. [file 12964_2024_1904_MOESM6_ESM.pdf]

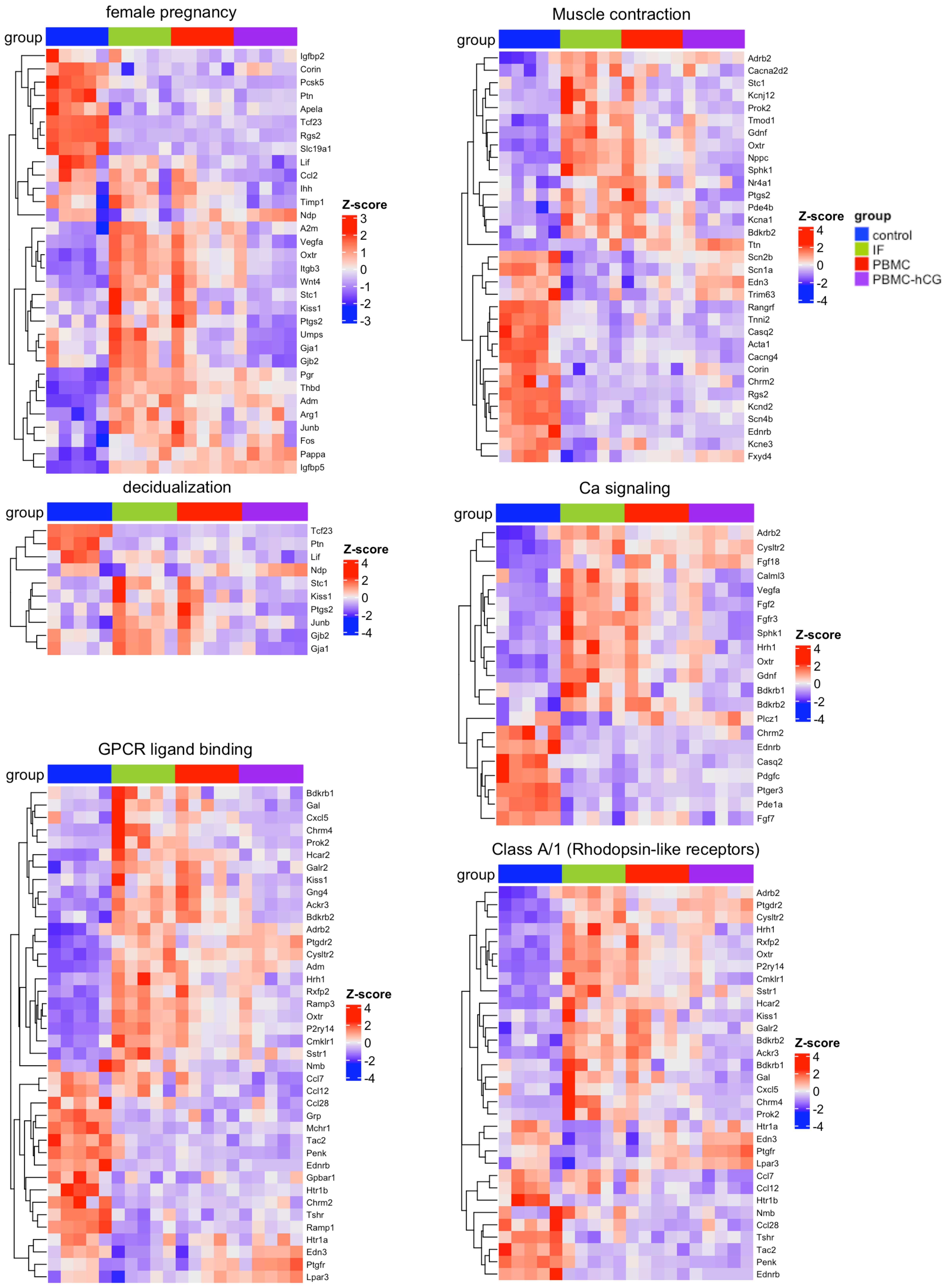
female pregnancy
group
Igfbp2
Corin
Pcsk5
Ptn
Apela
Tcf23
Rgs2
Slc19a1
Lif
Ccl2
Ihh
Timp1
Ndp
A2m
Vegfa
Oxtr
Itgb3
Wnt4
Stc1
Kiss1
Ptgs2
Umps
Gja1
Gjb2
Pgr
Thbd
Adm
Arg1
Junb
Fos
Pappa
Igfbp5
Z-score
3
2
1
0
-1
-2
-3
Muscle contraction
group
Adrb2
Cacna2d2
Stc1
Kcnj12
Prok2
Tmod1
Gdnf
Oxtr
Nppc
Sphk1
Nr4a1
Ptgs2
Pde4b
Kcna1
Bdkrb2
Ttn
Scn2b
Scn1a
Edn3
Trim63
Rangrf
Tnni2
Casq2
Acta1
Cacng4
Corin
Chrm2
Rgs2
Kcnd2
Scn4b
Ednrb
Kcne3
Fxyd4
Z-score
4
2
0
-2
-4
group
control
IF
PBMC
PBMC-hCG
decidualization
group
Tcf23
Ptn
Lif
Ndp
Stc1
Kiss1
Ptgs2
Junb
Gjb2
Gja1
Z-score
4
2
0
-2
-4
Ca signaling
group
Adrb2
Cysltr2
Fgf18
Calml3
Vegfa
Fgf2
Fgfr3
Sphk1
Hrh1
Oxtr
Gdnf
Bdkrb1
Bdkrb2
Plcz1
Chrm2
Ednrb
Casq2
Pdgfc
Ptger3
Pde1a
Fgf7
Z-score
4
2
0
-2
-4
GPCR ligand binding
group
Bdkrb1
Gal
Cxcl5
Chrm4
Prok2
Hcar2
Galr2
Kiss1
Gng4
Ackr3
Bdkrb2
Adrb2
Ptgdr2
Cysltr2
Adm
Hrh1
Rxfp2
Ramp3
Oxtr
P2ry14
Cmklr1
Sstr1
Nmb
Ccl7
Ccl12
Ccl28
Grp
Mchr1
Tac2
Penk
Ednrb
Gpbar1
Htr1b
Chrm2
Tshr
Ramp1
Htr1a
Edn3
Ptgfr
Lpar3
Z-score
4
2
0
-2
-4
Class A/1 (Rhodopsin-like receptors)
group
Adrb2
Ptgdr2
Cysltr2
Hrh1
Rxfp2
Oxtr
P2ry14
Cmklr1
Sstr1
Hcar2
Kiss1
Galr2
Bdkrb2
Ackr3
Bdkrb1
Gal
Cxcl5
Chrm4
Prok2
Htr1a
Edn3
Ptgfr
Lpar3
Ccl7
Ccl12
Htr1b
Nmb
Ccl28
Tshr
Tac2
Penk
Ednrb
Z-score
4
2
0
-2
-4
